# Multilayer Coatings for Tribology: A Mini Review

**DOI:** 10.3390/nano12091388

**Published:** 2022-04-19

**Authors:** Yanfei Liu, Shengtao Yu, Qiuyu Shi, Xiangyu Ge, Wenzhong Wang

**Affiliations:** 1 School of Mechanical Engineering, Beijing Institute of Technology, Beijing 100081, China; liuyanfei@bit.edu.cn (Y.L.); 3120210435@bit.edu.cn (S.Y.); wangwzhong@bit.edu.cn (W.W.); 2 State Grid Smart Grid Research Institute Co., Ltd., Beijing 102202, China; shiqiuyu@geiri.sgcc.com.cn

**Keywords:** multilayer coating, friction, wear, tribology, transition-metal nitride, diamond-like carbon

## Abstract

Friction and wear usually lead to huge energy loss and failure of machine pairs, which usually causes great economic losses. Researchers have made great efforts to reduce energy dissipation and enhance durability through advanced lubrication technologies. Single-layer coatings have been applied in many sectors of engineering, but the performance of single-layer coatings still has many limitations. One solution to overcome these limitations is to use a multilayer coating that combines different components with varied physical and chemical properties. In addition, multilayer coating with alternating layers only containing two components can lead to improved performance compared to a coating with only two different layers. This paper systematically reviews the design concept and properties of different types of multilayer coatings, including transition-metal nitride coatings, diamond-like carbon-based coatings, and other multilayer coatings. The inherent functional mechanisms of the multilayer structures are also detailed and discussed.

## 1. Introduction

Friction and wear occur in moving pairs with direct contact among all mechanical systems, leading to excessive energy consumption and failure of equipment [1]. Advanced techniques have been proposed to reduce friction and wear [2,3]. One of the methods is to deposit coating materials on friction pairs, which has been widely used for a long time due to its high performance in practical engineering applications. Coatings can be designed with different materials and structures to provide multiple functions. With the development of coating systems, different designing procedures have been proposed to further enhance the performance of coatings, or to extend the adaptivity of coatings in various environments [4,5,6]. One of the strategies is the multilayer designing of coating systems. Among the coating design concepts, multilayer coatings have attracted a lot of attention because the properties including hardness, elastic modulus, lubrication performance, and adhesion to substrate can be targeted and regulated, making it easier to develop coating systems to meet specific requirements.

In this paper, the design concepts and properties of different types of multilayer coatings, including transition-metal nitride (TMN) coatings, DLC-based coatings, and other multilayer coatings, are systematically reviewed. The inherent functional mechanisms of the multilayer structures are also detailed and discussed.

## 2. Development of Multilayer TMN Coatings

The failure of tools can lead to great costs induced by the stopping of production and new adjustment of machines. Around 10% of the losses can be reduced through optimizing the lifetime of finishing parts [7]. Decreasing the wear of tools, better control of the forming process, and a reduction in lubricant and cleaning agents are the motivations to promote the development of coatings. Chemical vapor deposition (CVD) and physical vapor deposition (PVD) are commonly used techniques for the fabrication of coatings, where CVD coatings usually have superior properties compared to PVD coatings due to the higher deposition temperature. However, CVD coatings have higher cost and more time consumption, but limited friction-reduction performance [8]. Attributed to the high performance and relatively low cost, PVD coatings are a promising alternative of CVD coatings for wider industrial applications.

The transition-metal nitride (TMN) coatings are widely used in engineering applications due to their superior physical and chemical properties, including high hardness, wear resistance, thermal stability, and excellent corrosion and oxidation resistances [9,10,11,12]. TMN coatings are used as protective layers for cutting tools, molds, dies, or for abrasion and corrosion resistance in various fields, including aerospace, automotive, etc. The performances of several TMN coatings, including TiN [13,14,15,16], CrN [17,18,19,20], ZrN [21,22,23,24], MoN [25,26,27], NbN [28,29,30], and TaN [31,32], have been investigated for many years. The continuous development of advanced coating materials is motivated by the increased demands for industrial applications. It was showed that multilayer structure design can effectively improve the mechanical, chemical, as well as tribological properties of coatings [33]. In the multilayer coating, each layer exhibits a specific property, such as a thermal and diffusion barrier, adhesion to substrate, load carrying, lubrication, or wear resistance. The investigation of multilayer PVD coatings started in the 1970s [34,35] and was based on the models proposed by Koehler [36]. The model indicates that materials with high yield strength could be fabricated through alternating thin layers with different shear modules due to the inhibition of dislocation formation and mobility. The Al/Al_x_O_y_ coatings were deposited, and the performances of the coatings were investigated. It was found that based on the layer spacing, a Hall–Petch-type relationship was obeyed for yield stress [34]. Bunshah et al. also studied both the metal/ceramic [37] and metal/metal [38] coatings deposited with evaporation techniques. In general, mechanical properties of the coatings can be improved with decreased layer thickness.

Holleck et al. fabricated multilayer coatings with different ceramic materials, demonstrating that the improvements in adhesion, indentation toughness, hardness, and wear-resistance performance can be achieved with optimized layer thickness [39]. The improved performance of multilayer coatings is believed to partially attribute to the stress relaxation and crack deflection, which exists in various contact conditions with cyclic loading and fatigue. Besides the influence of yield strength, the stacking sequence also has influence on the coating performance. It was found that the alternation of layers with high/low shear modulus can provide more benefits for multilayer DLC/metal carbide coatings [40] or TiN/Ti coatings [41,42].

To further investigate the influence of the stacking sequence of multilayer coatings, it is effective to consider the coatings under a point or distributed load, causing the deflection of coatings and the deformation of substrates [33]. Under such a circumstance, the maximum stress would increase with increased coating thickness if considering the bending stress. If the coatings are isolated as individuals to exclude the influence of substrate, each layer in multilayer coatings has much less stress compared to that of the thick layer if each layer can slide over each other. Hence, the alternating of hard/soft layers can offer a shear zone to prevent the fracture of hard and brittle layers under deflection induced by applied load. Moreover, there are also some other influences of the layer thickness. Since the layers also need to support the normal load, the minimum thickness of the soft layers is limited to provide adequate support to the hard layers. Additionally, the increased layer thickness will lead to increased relative sliding distance between each layer. Incorporating many thin layers is an ideal way to ensure the load-support properties. The layer thickness also depends on the loading condition and the aimed application of the multilayer coatings. For example, larger coating thickness is usually needed with the presence of hard and coarse third bodies. The benefits of the structure consisting of layers with relatively high hardness and relatively low hardness were also revealed via cyclic impact test [43,44]. The wear of coatings induced by plastic deformation can be suppressed through the composition of soft and hard layers, indicating that multilayer coatings have better prospect as a solution for various engineering problems. The concept and mechanisms of multilayer coatings with distinct performances were introduced [45,46]. Even a simple two-layer structure exhibited better wear- and corrosion-resistance properties [47,48]. The theoretical investigations have great value for better structural optimization of the multilayer coatings.

With the main elements of the coatings, TMN coatings can be divided into Ti-based TMN coatings, Cr-based TMN coatings, and TMN coatings with other elements. Ti and Cr are widely used as adhesion layer between the multilayer coating and metal substrates, which can be attributed to that the high binding energy makes it easier to form carbides, nitrides, and oxides on the adhesion layer. However, the performance of multilayer coatings strongly depends on the structure [49], which should be carefully decided for the designing of multilayer coatings. Usually, the multilayer coatings tend to achieve low friction, high wear resistance, good adhesion, and suppressed cracking during friction process. Various multilayer coatings have been designed, and the inherent functional mechanisms were also studied.

### 2.1. Ti-Based Multilayer TMN Coatings

TMN coatings have been widely used due to their high hardness and excellent corrosion-resistance and wear-resistance behaviors [50,51]. However, the thickness and the service life of TMN coatings are largely restricted by the residual stress induced by coating fabrication and the brittleness of the coatings [52,53]. Designing multilayer coatings combining TMN layers and metallic layers is an effective strategy to enhance the toughness of the coating and to reduce internal stress [52,54]. Cheng et al. investigated the influence of thickness of the Ti layer on the crystalline structure and internal stress of the multilayer TiN/Ti coatings [55]. It was found that the internal stress between Ti layers and TiN layers, as well as the internal stress of the TiN/Ti coating, can both be decreased by the increased layer thickness of Ti. In addition, the crystallinity of Ti and TiN phased can be also increased, and the lattice strain can be decreased with the increased layer thickness of Ti.

**Figure 1:**
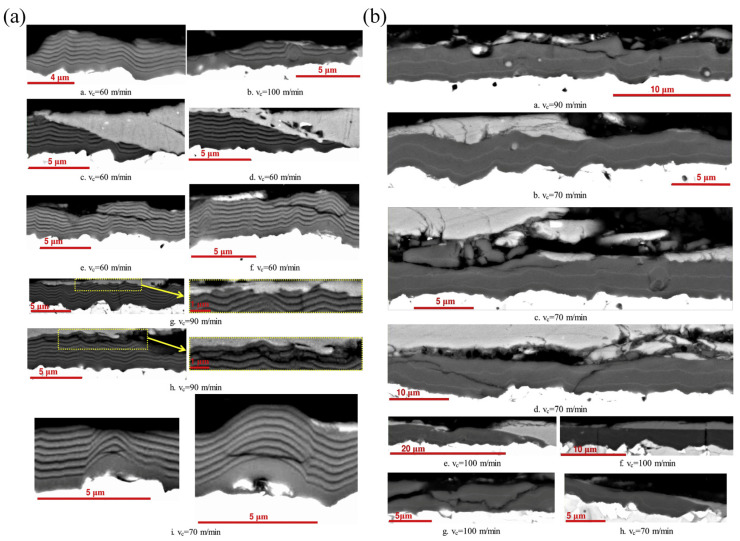
The fracture pattern on the Ti-TiN-(Ti,Al,Cr)N multilayer coatings with nanolayer thickness of (**a**) 302 nm and (**b**) 10 nm after the cutting tests with different cutting speeds (v_c_). (**a**) With nanolayer thickness of 302 nm, the formation of longitudinal cracks and internanolayer delaminations is typical. (**b**) With nanolayer thickness of 10 nm, rarer internanolayer delaminations can be observed. Reprinted with permission from [59].

The influence of the layer thickness of Ti on the mechanical and tribological performances of the multilayer coatings was further studied [56]. It was found that the increased layer thickness of Ti from 0 to 150 nm led to a reduced effective hardness; and the wear rate and plasticity of the multilayer coating were also increased, where the lowest COF was achieved with the 25 nm layer thickness of Ti. Bemporad et al. [52] also found that decreased hardness and wear-resistance performance can be caused by increased Ti/TiN ratio. Cheng et al. [57] investigated the influence of layer parameters of multilayer Ti/TiN coatings on the wear-resistance behavior, finding that the denser coating can be obtained with increased layer number, while the defects in the coating can be also reduced with increased layer number. However, the wear volume of the six-period multilayer coating was higher (more than 2 times) than that of the monolayer coating. The tribological properties of the multilayer TiN/Ti coatings were also investigated by Lackner et al. [58]. They found that the 16-bilayer coating with a bilayer period of 62 nm had the highest hardness, which could be attributed to the Hall–Petch strengthening mechanism. Furthermore, the multilayer coating with a high ratio of TiN/Ti had higher hardness, and the wear-resistance performance was also improved. Vereschaka et al. [59] investigated the influence of the layer thickness of the multilayer Ti-TiN-(Ti, Al, Cr)N coatings on the COF under temperatures from 500 to 1000 °C. It was found that the coating with 16 nm nanolayer thickness had the lowest COF and wear rate. With low layer thicknesses of 10 nm and 16 nm, delamination between nanolayers can be significantly suppressed (Figure 1). However, brittle fracture in the surface layers and the longitudinal cracks can be observed for the coating with a layer thickness of 16 nm. The lower COF of the multilayer coating can be attributed to that the machined material is separated from the coating surface. Azushima et al. also found that the grain orientation of TiN had significant influence on the COF of the coating [60]. The (111) preferred grain orientation exhibited lower COF comparing to that with (200) preferred grain orientation. Ghasemi et al. compared the tribological performance of monolayer TiN coating and multilayer Ti/TiN coating, finding that the multilayer coating exhibited lower COF compared to monolayer TiN coating, which can be attributed to the low shear strength of the Ti layer making it act as a lubricant of the multilayer coating during the friction test. The tribochemical reaction is also believed to have influence on the lubrication behavior, where the TiN_x_O_y_ and TiAlN_x_O_y_ tribofilm might be formed on the coating surfaces [61].

### 2.2. Cr-Based Multilayer TMN Coatings

Besides Ti-based coatings, Cr-based coatings have been also widely used in industrial applications [62,63]. Comparing to TiN coatings, CrN coatings are softer, less brittle and have less stress [64]. TiN coatings present relatively high mechanical properties and thermal stability. However, TiN coatings can be easily oxidized into TiO_2_ when the temperature is higher than 500 °C, leading to crack formation in the TiN coatings [65,66]. Differently, CrN coatings have better oxidation-resistance performance due to the formed dense Cr_2_O_3_ layer on the coating surface as protection layer [67,68]. Previous research also indicated that CrN coatings exhibited lower COF [69] and wear rate [70] compared to TiN coatings. However, when exposed to high temperatures up to 800 °C, the mechanical performance of the CrN coatings would deteriorate due to the loss of N [71,72]. Du et al. found that with the multilayer structure design of TiN/CrN coatings, the oxidation of CrN can be suppressed because that the TiN layer has higher binding energy than CrN, leading to better thermal stability and oxidation-resistance performance of the multilayer coating [73].

**Figure 2:**
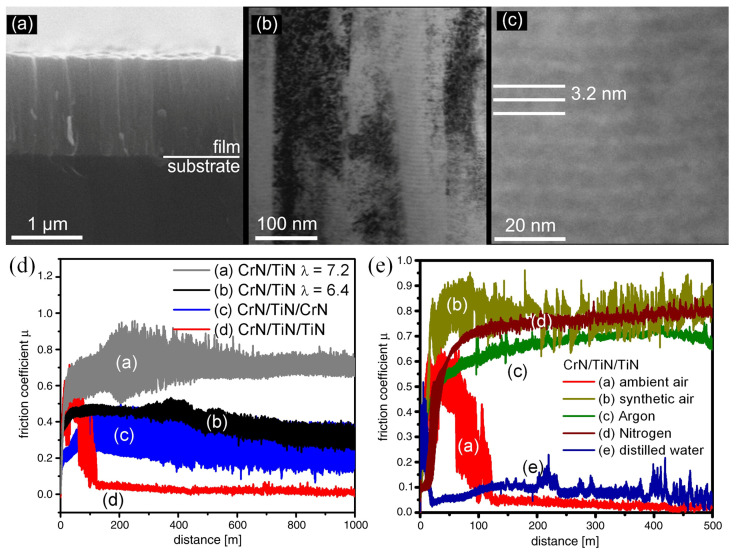
(**a**) SEM and (**b**,**c**) TEM image of multilayer CrN/TiN coating with bilayer period of 6.4 nm; (**d**) COF of different multilayer coatings under ambient condition; (**e**) COF of CrN/TiN/TiN coating under different environment conditions. Reprinted with permission from [78].

In the later studies, it was found that multilayered TiN/CrN coatings have superior properties compared to both homogenous TiN and CrN coatings [74,75]. It was shown that a low layer thickness, especially for the CrN layer in the multilayer coating, should be achieved to obtain better mechanical and tribological properties of the multilayer coatings. Zhou et al. investigated the tribological performances of TiN/CrN multilayer coatings [76]. They found that the TiN coating had a higher COF of 0.9, while the TiN/CrN multilayer coatings had a lower COF of 0.3–0.5. Meanwhile, the TiN/CrN multilayer coatings also had a lower wear rate comparing to TiN. The reduction in COF and wear rate of TiN/CrN multilayer coatings can be attributed to the enhanced hardness, the formation of a dense oxidation layer with CrO_3_ and Cr_2_O_3_, and the exclusion of the third-body particles from the wear regions. Srinivasan et al. found that the TiN/CrN multilayer coatings deposited on hard substrate had a lower wear rate comparing to that of TiN and Ti/TiN multilayer coating at both room temperature and high temperature [77]. Paulitsch et al. investigated the tribological performances of CrN/TiN multilayer coatings in different atmospheres (Figure 2) [78]. It was found that the multilayer CrN/TiN/TiN coatings had a steady-state friction coefficient of 0.05 in ambient air (RH ≈ 25%), whereas they had a much higher COF between 0.65–0.75 in Ar, N_2_ and synthetic air with low humidity (RH < 1%). However, the wear rate in ambient air was higher than that in Ar and N_2_ atmospheres. These results suggested that the COF can be increased in a nonoxidizing environment due to the absent of oxidants as lubricant, while the wear-resistance performance can be enhanced due to the suppressed oxidation and fewer third-body particles. However, the COF varied significantly with the arrangement of different layers. With CrN/TiN/CrN coating, the COF can be reduced to 0.25. However, when the CrN/TiN/CrN was replaced with CrN/TiN/TiN, the COF significantly reduced to 0.05. The influence of the structure of the multilayer coating and the tribochemical reaction on the lubrication performance still need further investigation.

### 2.3. TMN Coatings with Other Elements

**Figure 3:**
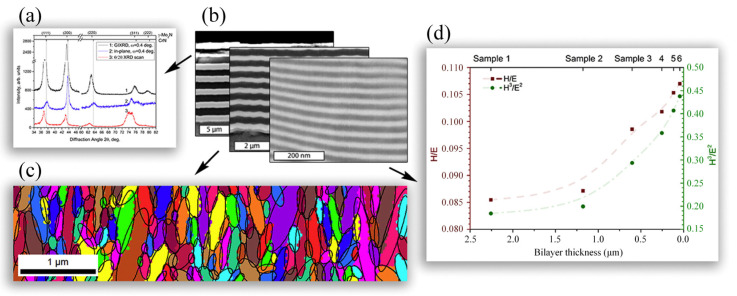
Multilayer designing of CrN/MoN coatings. (**a**) XRD results and (**b**) cross-section images of CrN/MoN coatings with different layer thicknesses; (**c**) EBSD results of a CrN/MoN coating; (**d**) mechanical behavior of CrN/MoN coatings with different layer thicknesses. Reprinted with permission from [83].

ZrN coatings have attracted attention in several industrial applications due to outstanding optical properties, excellent chemical stability, a high melting point [79], and excellent wear-resistance performance [80]. Researchers found that the wear-reduction property of ZrN coating can be further improved by the multilayer structure with Zr layers [81]. The influence of the thickness of the adhesion layer and wear-resistant layer on the adhesion strength, microhardness, and other performances of the ZrN-(Zr, Al, Si)N coatings was investigated by Vereschaka et al. [82], indicating that smaller thickness of the adhesion layer led to more active wear. Postolnyi and Pogrebnjak et al. [83,84] designed superhard protective multilayer CrN/MoN coatings with enhanced toughness and hardness through the arc-PVD technique for industrial applications (Figure 3). The element and phase composition, coating structure, mechanical properties, and the residual stress were detailed investigated. In addition, the influence of the deposition conditions was also studied. During the fabrication process, most of the parameters were fixed to keep the similar elemental and phase compositions, while the deposition time was changed to fabricate coatings with different layer thickness. With smaller layer thickness, the volume of interfaces and the layer number of interfaces both increased, leading to Hall–Petch strengthening of the multilayer coatings. In addition, the propagation of cracks and dislocations can be blocked in the multilayer coatings.

### 2.4. Doping with Different Elements

Doping different metal elements into TMN coatings is also a practical method to enhance their performances. Among those coatings, TiCrN coatings have attracted much attention due to the oxidation resistance at high temperature, high hardness, and low COF [85,86]. Nainaparampil et al. investigated the tribological behaviors of TiCrN coatings, where the major phases are CrN, TiN, and Cr_2_N. TiCrN coatings exhibited lower COF compared to TiN and CrN coatings [87]. It was found that the plastic deformation of wear debris formed during the friction process is the dominant reason for the low COF. Ezazi et al. [86] compared the mechanical performances and tribological behaviors of magnetron-sputtered Cr/CrN, Ti/TiN, and TiCr/TiCrN coatings on aerospace aluminum. Different coatings exhibited varied behaviors, where the Ti/TiN coating had the highest wear resistance performance; the Cr/CrN coating had the highest surface hardness; and the TiCr/TiCrN coating had the smoothest surface and lowest COF among all the test samples. The functional mechanisms of the coatings were also investigated. The Cr/CrN coating suffered more severe wear due to the oxidative abrasive wear; the brittle fracture wear of Ti/TiN coating was attributed to the slight plastic deformation; the mixed phases with CrN and TiN formed by TiCr/TiCrN coating during the friction process led to an intermediate wear-reduction performance.

The influence of element concentration and microstructure on the performance of Cr/CrN/CrTiN coatings was also investigated through various characterization techniques [88]. It was found that the increased Ti content in the coatings led to increased hardness and elastic modulus, and increased H/E and H^3^/E^2^ ratios. However, the increased Ti content in the coatings led to a decreased scratching toughness, which was attributed to the conformal cracking at higher load. In addition, the wear-resistance performance of the coatings decreased with increased Ti content, which was attributed to the mismatch between the modulus of coating and substrate. In general, they found that the multilayer structure can effectively reduce the residual stress compared to the monolayer coating, leading to higher adhesion stress and better wear-resistance performance.

Özkan et al. [89] investigated the mechanical and tribological behaviors of multilayer CrTiN/TiCN and CrTiN/CrCN coatings deposited by arc-PVD with different thicknesses. It was found that CrTiN/CrCN coatings exhibited excellent lubrication and wear-reduction performances under elevated contact pressures. The graphitization of the amorphous carbon phase of CrTiN/TiN + CrCN and CrTiN/TiN + TiCN coatings was believed to be the reason for the different lubrication performance. For CrCN coatings, the better graphitization and lower oxidation dominate the lubrication and wear-resistant behaviors. The results showed that the service life of tools and molds can be extended with deposited CrTiN/TiN + CrCN coatings even at high contact pressures. In addition, the performances can be further improved by multilayer CrTiN/TiN + CrCN/TiCN coating with thin interlayers and thick ceramic layers. Purushotham et al. [90] investigated the tribological performances of Zr-implanted TiN coatings, finding that the implantation of Zr led to decreased hardness of the coatings. The increased dose led to an increased layer thickness of the implanted zone, causing a reduced COF. The results were accordance with the model with a thin soft film on a harder surface. However, considering that the COF in this study can be surprisingly dropped from 0.8–0.9 to 0.1 with the implantation of Zr, the inherent lubrication mechanisms with implanted elements still need further investigation to illustrate the function of element doping. Previous research mainly focused on the mechanical and wear-resistance performances of TMN coatings, but limited research focused on the influence of structure and composition of coatings on the frictional behaviors. The frictional behaviors of TMN coatings are also important for engineering applications, which still need more investigation in the future.

## 3. Development of Multilayer DLC Coatings

**Figure 4:**
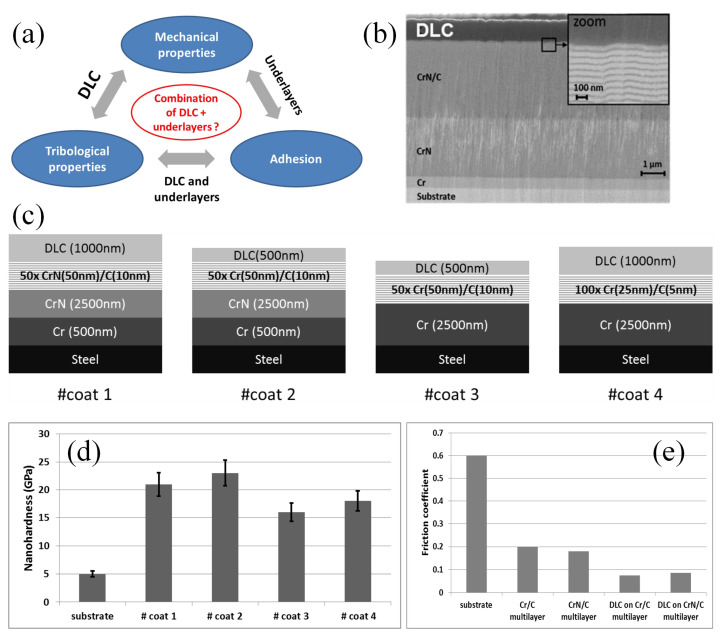
(**a**) Relationships between underlayers and the properties in a DLC-based multilayer coating; (**b**) the cross-sectional morphology of multilayer coating; (**c**) structure of multilayer coatings with different underlayers; (**d**) hardness of different multilayer coatings; (**e**) COF of different multilayer coatings. Reprinted with permission from [93].

Based on the concept of multilayer designing of coating systems, the multilayer ceramic metal-DLC coatings were fabricated by Voevodin et al. [45] using electron-enhanced unbalanced magnetron sputtering for sliding wear applications. Low COF and low wear rates can be achieved by the multilayer coatings with upper Ti20%-DLC and Ti35%-DLC layers. Sui et al. [91] prepared CrN/DLC/Cr-DLC multilayer coatings with plasma-enhanced chemical vapor deposition, which can significantly improve the lubrication and wear-resistance performance comparing to single component coating. The improved performances of the CrN/DLC/Cr-DLC multilayer coatings can be attributed to the lubrication of DLC layers, the supporting of CrN layers, the enhanced crack propagation inhibition, and the increased elastic recovery governed by the multilayer structure. DLC coatings were also combined with MoS_2_ to enhance the lubrication and wear-resistance performances. Pu et al. [92] prepared a multilayer DLC/MoS_2_ coating using medium-frequency magnetron sputtering, which exhibited a low COF of 0.02 and a low wear rate of ~6.5 × 10^−6^ mm^3^ N^−1^ m^−1^. The influence of different underlayers on the tribological behaviors of the DLC-based multilayer coatings prepared by magnetron sputtering was investigated by Duminica et al. (Figure 4) [93], where a better adhesion could be achieved with only Cr under layer, exhibiting lower COF compared to other samples.

For single-layer DLC coating, high residual stress would lead to brittle fracture and delamination under high normal load during the friction process. Researchers found that the tribological behaviors of multilayer DLC coatings can be improved through the DLC layers with different properties. Li et al. [94] fabricated multilayer DLC coatings with alternated soft and hard layers through the alternating of bias during magnetron sputtering. Delamination was observed in monolayer coatings due to high residual stress. The results showed that the bonding structure (sp^3^ and sp^2^) can be changed by substrate bias. The sp^3^ fraction in DLC coating can be increased with increased bias ratio on the two adjacent sublayers from −40 V/−160 V to −80 V/−160 V, leading to increased coating hardness. With the multilayer designing, the hardness of multilayer DLC coating was similar to the coatings deposited at low constant bias, but the adhesion strength and toughness were significantly improved. It can be concluded that alternately biased sputtering deposition is a promising way to fabricate DLC coating with high hardness, toughness, and adhesion strength. With the similar designing concept, Harigai et al. [95] fabricated multilayer N-DLC coatings with each layer thickness of 10 nm using filtered arc deposition, containing periodic bilayer structures with ta-C:N and soft a-C:N layers. The multilayer coatings showed better wear-resistance performance than monolayer ta-C:N coating and multilayer N-DLC coatings with each layer thickness of 50 nm. Lin et al. [96] fabricated multilayer DLC coatings with alternated soft and hard layers using unbalanced closed-field magnetron sputtering to enhance wear-resistance performance at high contact stress. It was found that the multilayer coating with a soft top layer had lower wear volume under high contact stress, which can be attributed to the fact that the soft top layer can form a transfer layer to reduce friction and wear.

## 4. Other Multilayer Coatings for Tribology Applications

**Figure 5:**
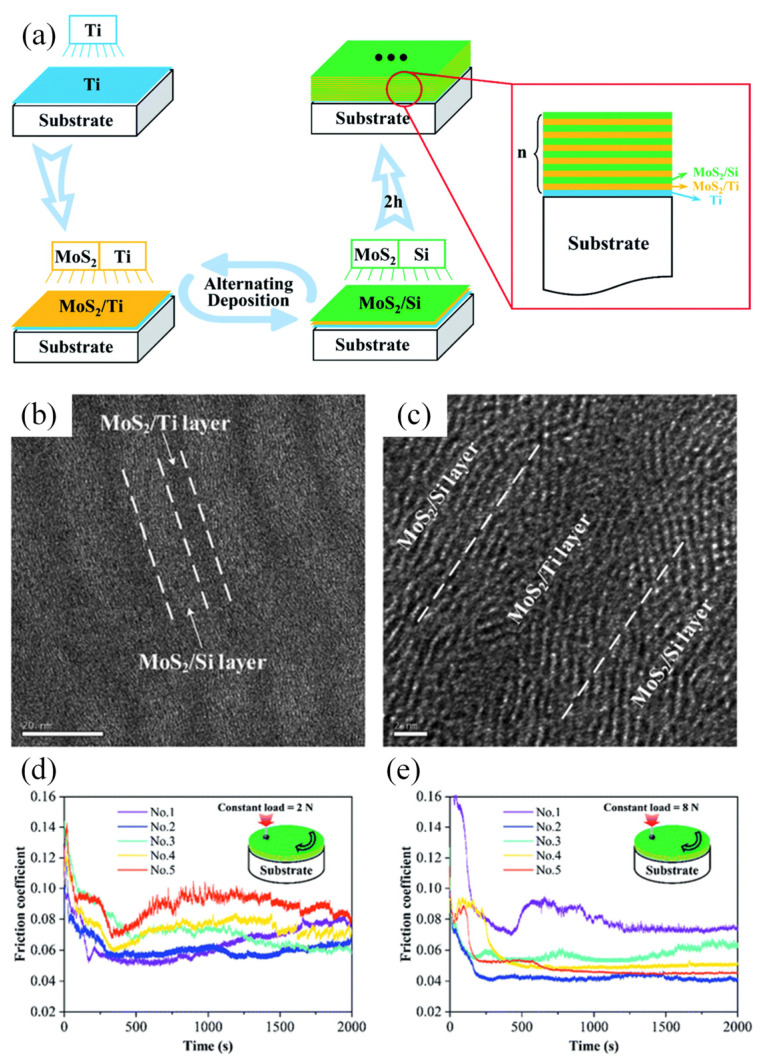
(**a**) Schematic diagram of the MoS_2_/Ti–MoS_2_/Si multilayer coating; (**b**,**c**) cross-sectional morphology of a MoS_2_/Ti–MoS_2_/Si multilayer coating; (**d**,**e**) COFs of different MoS_2_/Ti–MoS_2_/Si multilayer coatings under normal loads of 2 N and 8 N. Reprinted with permission from [103].

MoS_2_ coatings exhibit excellent lubrication performance under high dry or vacuum conditions due to the easy shear between lattice layers [97,98]. However, when rubbed in humid air, the dangling bonds at the edge of MoS_2_ react strongly with O, resulting in higher COF and shorter service life [99,100,101]. Aiming to the shortcomings, MoS_2_-based multilayer coatings have been designed to further enhance the performance. The tribological behaviors of multilayer coatings of MoS_2_ and metallic including Au, Ni, Pb or PbO were studied in humid air with 50% relative humidity, which exhibited lower and more stable COF compared to pure MoS_2_ coating [102]. The function mechanism of metal for the sputter-deposited metal–MoS_2_ multilayer coatings is believed to be the optimization of the MoS_2_ structure. Kong et al. [103] investigated the tribological behaviors of MoS_2_/Ti–MoS_2_/Si multilayer coatings deposited by magnetron sputtering (Figure 5), indicating that better lubrication performance can be achieved by the multilayer design of coatings. Those results indicated that the multilayer structures have potential to improve the tribological behaviors of conventional MoS_2_ coating, but the inherent mechanisms are still worth further investigation to guide the designing of MoS_2_-based coatings for future application.

**Figure 6:**
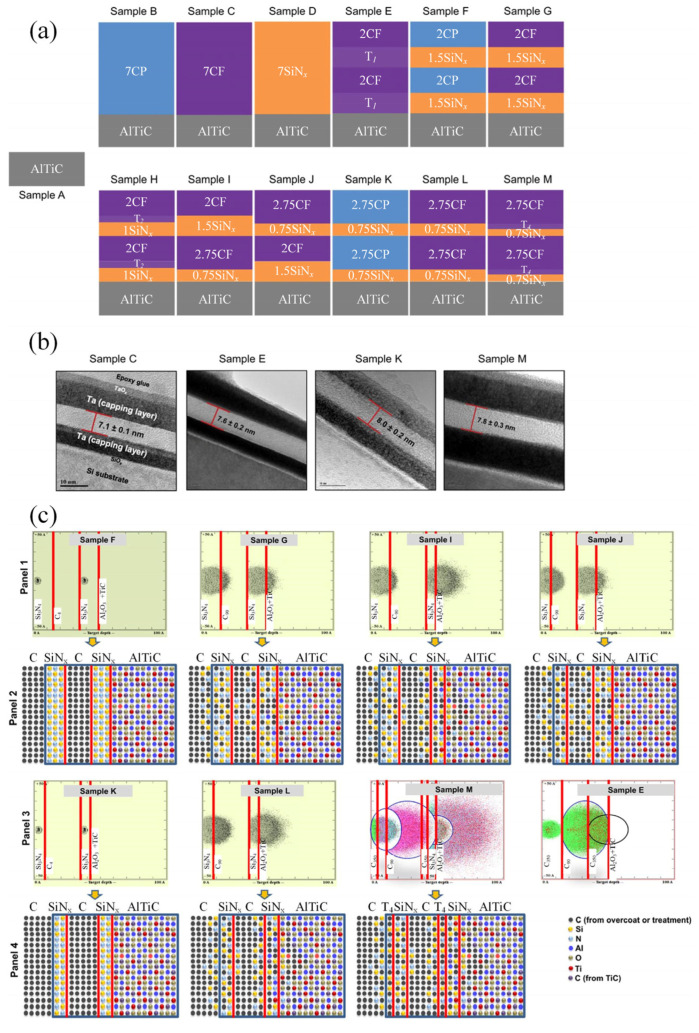
(**a**) Schematic diagram of the designing of coating samples; (**b**) cross-sectional images of the coatings; (**c**) TRIM simulations of the selected coating samples, showing the extent of atomic mixing. In this figure, AlTiC represents Al_2_O_3_ + TiC composite material; CP represents sputter-deposited carbon overcoat; CF represents FCVA-deposited carbon overcoat; T represents high-energy carbon treatment. Reprinted with permission from [106].

**Figure 7:**
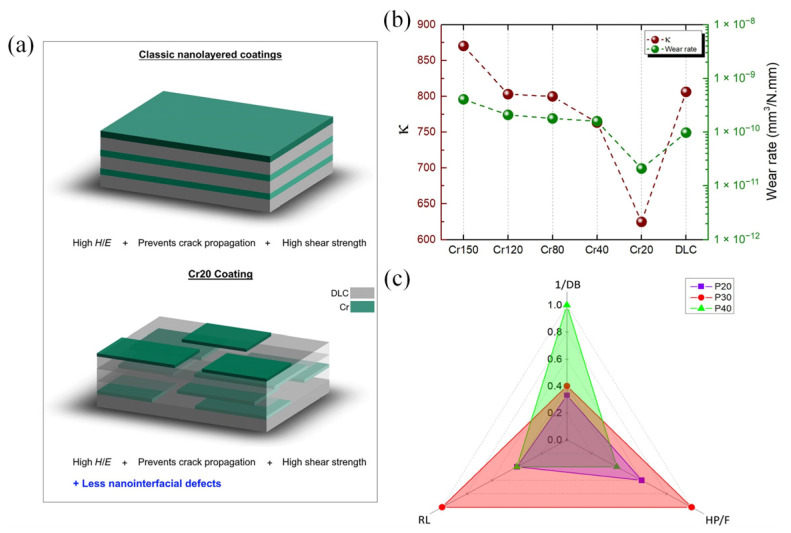
(**a**) Schematic diagram of the conventional multilayer coating and Cr20 coating; (**b**) relation between the wear rate and the proposed Ƙ parameter; (**c**) characteristics of the parameters affecting the tribological performance of the textured coating samples. Reprinted with permission from [107].

With the development of coating fabrication and characterization techniques, several new findings shed the light on the precision structure design of multilayer coatings from an atomic view [104,105]. Dwivedi et al. [106] developed C/SiN_x_ multilayer coatings with layer thickness of 7–8 nm using an enhanced atomic intermixing (formation of nanocomposite interfaces) approach (Figure 6), leading to 2–10 times better macroscale wear durability compared to conventional coatings with larger thickness of 20–100 nm. The enhanced performance can be attributed to the high sp^3^ bonding of the carbon overcoat and increased interfacial strength induced by intermixing, leading to improved adhesion and robustness of the coatings. Khadem et al. [107] designed discreate periodic nanolayered coatings, which had a different structure compared to conventional multilayer coatings (Figure 7). The discrete periodic nanolayered coatings exhibited better wear-reduction performance compared to conventional multilayer coatings, which can be attributed to the reduced interfacial defects. The tribological performance was further improved by surface-texturing treatment. Advanced research tools make it possible to investigate the fundamental mechanisms of multilayer coatings in tribological application.

**Figure 8:**
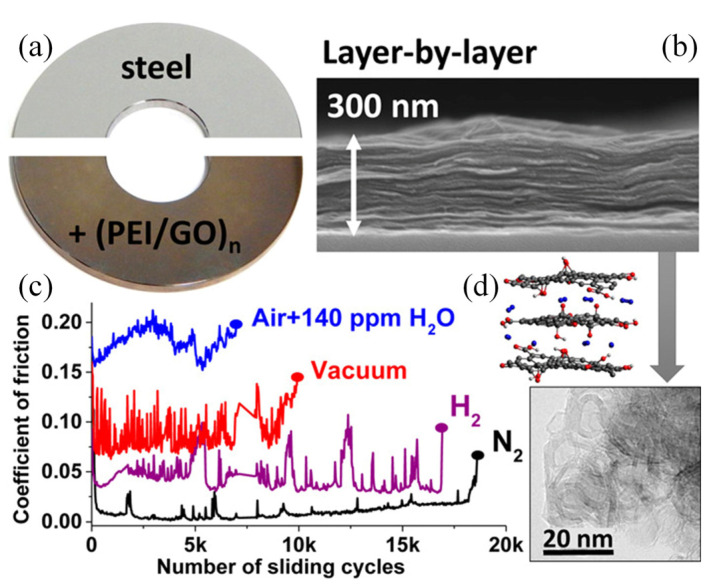
Multilayer design of PEI/GO coatings. (**a**) Photograph of the steel substrate and that deposited with multilayer PEI/GO coatings; (**b**) cross-sectional morphology of a PEI/GO coating with 15 bilayer periods; (**c**) COFs of the multilayer coatings under different test environments; (**d**) HRTEM image of the wear debris containing GO in N_2_ environment. Reprinted with permission from [124].

Two-dimensional (2D) materials, including graphene-family materials [108,109,110,111,112,113], MoS_2_ [99,100], and black phosphorus [114,115,116,117] have been used as lubricants because of their low interlayer shear strength. Recently, multilayer coatings with 2D materials have also been designed to promote tribological properties. Most recently, Fan et al. [118,119,120,121,122,123] fabricated coatings with Ti_3_C_2_T_x_ Mxene and achieved excellent self-healing, antiwear, and anticorrosion capacity. Saravanan et al. [124] fabricated multilayer coatings with graphene oxide and PEI via layer-by-layer assembly technique (Figure 8). Macroscale superlubricity (COF < 0.01) can be achieved with the multilayer coating having a thickness of about 300 nm. The superlubricity mechanism is believed to be the formation of carbon nanoparticles in dry conditions. In the subsequent study, it was found that the formation of transfer layer is also critical for the achieving of ultralow friction [125]. Achieving macroscale superlubricity is possible with multilayer coatings containing 2D materials, but the environment adaptivity still needs to be improved, and the inherent mechanisms also need to be further investigated.

## 5. Mechanisms for Controlling Friction and Wear Using Multilayer Coatings

**Figure 9:**
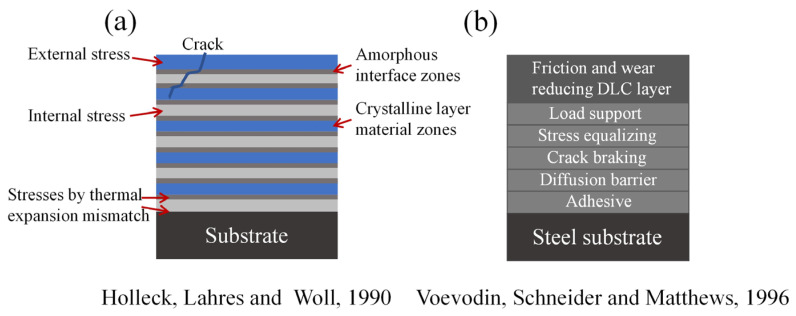
Schematic diagram of the mechanisms of the multilayer designing of coating. (**a**) Crack deflection and stress relaxation in multilayer coating. Reprinted with permission from [39]. (**b**) Theoretical constitution of supporting interlayers of DLC coating. Reprinted with permission from [45].

Coatings have been widely used in industrial applications as protection for cutting tools, dies, pistons, etc. However, the performance of monolayer coatings is usually restricted by their poor adhesion with substrate, and the high residual stress induced by the fabrication process. In addition, the mismatch of the mechanical properties between substrates and coatings also suppresses the performance of monolayer coatings. Aiming to solve these problems, the concept of multilayer coating has been proposed; and lots of work has been carried out to enhance the coating performance through the multilayer structure. One of the fundamental concepts of the multilayer design is stress relaxing and crack deflection (Figure 9a) [39]. Back in the 1970s–80s, researchers attempted to build multilayer coatings through alternating thin layers with high-shear-modulus and thin layers with low shear modulus based on the models of Hoehler. In 1990, Holleck et al. [39] found that the multilayer structure of TiC/TiB_2_ coatings leads to the deflection of cracks through the interface zones, causing energy dissipation without coating failure. Layer thickness also has influence on the stress distribution of the multilayer coatings. In addition, from an engineering point of view, when a normal force is applied on the coating’s surface, the multilayer coating with thin, soft layers can reduce the maximum bending stress. With the multilayer design with soft and hard layers, the plastic yielding of hard layers can be avoided, especially under the condition with cyclic loading and fatigue. Another fundamental concept for the designing of multilayer coating is the functional design of different layers for purposes such as adhesion, load supporting, lubrication, and wear reduction, etc. (Figure 9b) [45]. The wear-resistance and lubrication performances can be enhanced through the multilayer design of the coatings. However, macroscale friction is a complex physical–chemical process. The friction and wear-reduction mechanisms of multilayer coatings with different structures and compositions are different. Hence, various mechanisms for controlling friction and wear using multilayer coatings have been proposed (Table 1), which can guide the future development of the multilayer coating systems.

## 6. Conclusions and Perspectives

This paper reviewed the multilayer structure designing of different types of coatings. With the development of multilayer coatings, the influence of various parameters, including the layer thickness, element composition, etc., and the influence of supporting layers on the behaviors of top layer have been systematically investigated. However, in the view of the authors, there are still many unsolved problems for further investigation. Many studies reported that the coatings with multilayer structures have much lower COF compared to monolayer coatings or multilayer coatings with different structures. However, the underlying mechanisms still need further investigation with a view to guide future high-performance multilayer coatings. In addition, advanced multiphysics simulation works are also needed for the optimization of material and structure design of multilayer coatings. Recent years, multilayer coatings containing 2D materials have also been designed [124], which achieved macroscale superlubricity under dry atmosphere. Multilayer coatings with 2D materials sometimes exhibit excellent tribological behaviors. However, related studies are restricted to limited types of 2D materials. The influence of coating structure, chemical composition of 2D materials or the matrix on the tribological behaviors still needs further investigation for the development of novel lubrication systems.

## Figures and Tables

**Table 1:** Friction and wear-reduction mechanisms of multilayer coatings.

Types of Multilayer Coatings	Preparing Methods	Lubrication Properties	Friction-Reduction Mechanisms	Wear-Reduction Mechanisms
TiN/Ti [56]	Large area filtered cathodic arc deposition	COF reduced from 0.82 (TiN) to 0.6 (with Ti layers thickness of 25 nm)	Lower shear strength of soft Ti layers	—
Ti/TiN [61]	High-vacuum magnetron sputtering	COF reduced from 0.54 (TiN) to 0.48	Formation of TiAlN_x_O_y_ and TiN_x_O_y_ tribolayers	—
TiN/CrN [76]	Reactive magnetron cathodic sputtering	COF reduced from 0.9 (TiN) and 0.6–0.7 (CrN) to 0.3–0.5	Enhanced hardness and formation of the dense Cr_2_O_3_, and CrO_3_ oxide layer	
CrN_HIPIMS_/TiN_DCMS_ [78] *	High-power impulse magnetron sputtering (HIPIMS) and DC unbalanced magnetron sputtering (DCMS)	COF reduced to 0.05	Formation of humidity-triggered layers during dry-sliding tests under humid conditions	—
(Ti–Cr)N [87]	Cathodic arc deposition	COF reduced from 0.7 (TiN) and 0.75 (CrN) to 0.4	Formation of mixed-phase films with plastic deformed wear debris	
CrTiN/TiCN and CrTiN/CrCN [89]	Cathodic arc PVD	COF reduced from 0.8–1.0 (bare substrates) to 0.2	Graphitization of the amorphous carbon phase	Improved adhesion between individual layers; increased coating hardness; graphitization
CrN/DLC/Cr-DLC [91]	PECVD	COF reduced to 0.087	Lubrication of DLC; supporting of CrN layers; enhancement of crack-propagation inhibition; increased elastic recovery capability	
Multilayer DLC with hard and soft layers [96]	Unbalanced closed-field magnetron sputtering	Lower COF during running-in process with soft top layer	Formation of transfer layer with soft top layer to provide low friction and wear	
MoS_2_/Ti–MoS_2_/Si [103]	Unbalanced magnetron sputtering	COF reduced to 0.0432	Improved compactness and orientation of MoS_2_; improved oxidation and moisture resistance of MoS_2_; higher hardness; hindered dislocations motion and crack propagation	
C/SiN_x_ overcoats [106]	Magnetron sputtering in situ with carbon deposition; high-energy carbon treatment	COF reduced from 0.4 (bare substrates) to lower than 0.2	Extremely high adhesion governed by atomic intermixing, sufficient carbon thickness; high sp^3^ bonding	
Polyethylenimine/graphene oxide [124] *	Layer-by-layer deposition	COF reduced from 0.60 (substrate) to lower than 0.01	Reduction in the contact area due to the formation of carbon nanoparticles in dry conditions	—

* The influence of environment humidity on the lubrication behaviors was investigated.

## Data Availability

Not applicable.
